# Adolescent loneliness and precarious employment in young adulthood: The moderating role of school attachment

**DOI:** 10.1111/jora.70266

**Published:** 2026-09-10

**Authors:** Hyewon Son, Kyungeun Song, Jinho Kim

**Affiliations:** ^1^ Department of Sociology The University of Texas at Austin Austin Texas USA; ^2^ School of Social Work University of Michigan Ann Arbor Michigan USA; ^3^ Department of Sociology University of Michigan Ann Arbor Michigan USA; ^4^ Department of Health Policy and Management Korea University Seoul Republic of Korea; ^5^ Interdisciplinary Program in Precision Public Health Korea University Seoul Republic of Korea

**Keywords:** adolescent loneliness, employment quality, life‐course perspective, precarious employment, school attachment

## Abstract

Young adults are increasingly exposed to precarious employment, yet little is known about how early‐life social experiences relate to labor‐market outcomes in adulthood. Drawing on life‐course perspectives, this study examines whether adolescent loneliness is associated with precarious employment in young adulthood and whether school and neighborhood attachment moderate this association. We used longitudinal data from the National Longitudinal Study of Adolescent to Adult Health. Adolescent loneliness was measured in Wave I, and precarious employment was assessed in young adulthood (Wave IV). To account for unobserved family‐level confounding, we employed sibling fixed effects models that compare siblings within the same family. Interaction terms were used to examine the moderating roles of school and neighborhood attachment. Adolescent loneliness was positively associated with higher levels of precarious employment in young adulthood. This association persisted after sibling fixed effects adjustment, indicating that it is not fully accounted for by shared family background. Supplementary analyses showed stronger associations with dimensions related to job stability, access to benefits, and workplace control. School attachment significantly moderated the association: the loneliness–precarity association was attenuated among adolescents with higher school attachment. Neighborhood attachment did not exhibit a significant moderating pattern. These findings suggest that adolescent loneliness is associated with enduring differences in employment quality in young adulthood and identify schools as key institutional contexts in which this association is attenuated. These findings point to adolescent loneliness and school belonging as important considerations for research and policy on inequality in young‐adult employment quality.

## INTRODUCTION

The transition from adolescence to adulthood has become increasingly uncertain as stable, long‐term employment has grown less accessible for younger cohorts. In the United States and other advanced economies, young adults are disproportionately concentrated in precarious forms of employment, characterized by unstable contracts, limited access to employment‐based benefits, low job security, and restricted workplace autonomy (Julià et al., 2017; Kalleberg, 2009). These labor market conditions reflect broader processes of economic restructuring and labor market flexibilization that have reshaped early career trajectories (Mills & Blossfeld, 2005). Importantly, precarious employment is not merely a temporary phase of labor market entry; rather, it has been linked to enduring socioeconomic disadvantage and cumulative exposure to employment instability (Gash, 2008). A growing body of evidence documents the adverse consequences of precarious employment for individual well‐being (Park et al., 2024). Precarious work is associated with elevated psychological distress, material hardship, and economic insecurity, as well as poorer self‐rated health and increased risk of chronic disease (Benach et al., 2014, 2016; Donnelly, 2022; Oddo et al., 2024). Despite extensive research on the structural and macroeconomic determinants of employment precarity, far less is known about how early‐life social experiences contribute to vulnerability to precarious employment in adulthood.

Adolescence represents a critical developmental period during which social relationships and institutional contexts play a central role in shaping long‐term socioeconomic trajectories. During this stage, peer interactions, school engagement, and perceptions of social belonging are closely tied to the development of social skills, self‐efficacy, and future‐oriented aspirations that have enduring implications for adult outcomes (Crosnoe & Johnson, 2011; Elder et al., 2003; Jang et al., 2026). Among these social experiences, loneliness has emerged as a particularly salient psychosocial risk factor (Cacioppo et al., 2015). Loneliness is the subjective experience of perceiving one's social relationships to be deficient relative to what one desires. It has been conceptualized as encompassing both a cognitive‐evaluative component, in which individuals appraise themselves as insufficiently accepted or valued by others, and an affective component, comprising the distressing feelings of isolation that accompany that appraisal (Hawkley & Cacioppo, 2010; Perlman & Peplau, 1981). Because loneliness concerns social connection, it might be assumed to operate through the same channels as social capital, the resources individuals derive from their social ties. The two constructs, however, are conceptually separable. Social capital is a structural and resource‐based property of social networks, referring to the benefits that flow from network position and membership (Coleman, 1988; Lin, 2001; Putnam, 2000), whereas loneliness is a subjective evaluation of the adequacy of one's relationships. The distinction matters because the two need not coincide. An adolescent embedded in an objectively large and resource‐rich network can nonetheless feel lonely, and one with few ties may feel little loneliness. It is this subjective, perceptual dimension, rather than the structural features of an adolescent's network, that we foreground.

Extant research demonstrates that adolescent loneliness is associated with a range of adverse outcomes extending into adulthood, including poorer educational attainment, heightened psychological distress, and compromised physical health, even after accounting for family background and co‐occurring mental health conditions (Kim et al., 2025; Kwon, Nam, & Kim, 2025; Qualter et al., 2015). Despite this evidence, little empirical work has examined whether adolescent loneliness has lasting consequences for labor market outcomes, particularly employment precarity.

We situate the link between adolescent loneliness and later employment quality within a life‐course framework, which treats adolescence as a developmentally sensitive period whose social and emotional experiences carry forward into later socioeconomic attainment (Elder et al., 2003; Heckman, 2006). Within this framework, we argue that loneliness is associated with the transition into adult labor market roles primarily through subjective and psychological pathways. Repeated perceptions of being insufficiently accepted may lower relational self‐efficacy and attenuate the generalized trust that supports stable and cooperative employment relationships (Hawkley & Cacioppo, 2010), and loneliness is further associated with a heightened sensitivity to social threat (Spithoven et al., 2017). These orientations are not fixed dispositions, but adaptations formed through earlier social experience, and they may shape how young people approach interpersonally demanding work.

Whether these orientations translate into precarious employment, however, depends on the opportunities young people encounter rather than on individual dispositions alone. Loneliness in adolescence is associated with lower educational attainment (Benner, 2011; Matthews et al., 2023), leaving lonely adolescents with thinner credentials and fewer of the signals employers rely on when allocating workers to jobs (Kerckhoff, 2001). Through such selection processes, they may be channeled toward less stable and less protected jobs even when they actively seek stable work. Precarious employment, defined by unstable contracts, limited benefits, and reduced autonomy, is in this sense a property of the positions made available to workers as much as a reflection of the dispositions they bring (Benach et al., 2014; Kalleberg, 2009). As these psychological and structural disadvantages accumulate across the transition to adulthood, they may leave some young adults with fewer resources to secure stable, high‐quality employment (Stanton‐Salazar, 2010).

The association between adolescent loneliness and later employment precarity may vary across institutional contexts, as adolescents are embedded within schools and neighborhoods that differ in the social resources they provide. Loneliness is an individual‐level subjective state, whereas schools and neighborhoods are features of the social environment in which adolescents are situated. We therefore treat attachment to these contexts not as an alternative indicator of an adolescent's social connectedness, but as a contextual resource that may offset the specific deficits through which loneliness operates. A strong sense of belonging to school, for instance, can provide structure, supportive ties to teachers and adults, and a basis for competence and self‐efficacy that does not depend on the peer acceptance lonely adolescents feel they lack (Bond et al., 2007; McNeely & Falci, 2004). To the extent that loneliness undermines educational engagement and relational confidence, attachment to school may sustain precisely these resources and weaken the path from loneliness to later employment precarity.

Neighborhood attachment may buffer loneliness through a partly different route. Cohesive neighborhoods are marked by collective efficacy, the shared trust and willingness of residents to support and monitor one another, which for adolescents can mean access to caring adults, informal supervision, and a sense of communal belonging beyond the family and peer group (Sampson et al., 1997; Wright & Fitzpatrick, 2006; Zimmerman et al., 2016). These resources may be especially valuable when peer relationships feel inadequate, and such embeddedness can also expose adolescents to informal information and role models relevant to the transition to work. Neighborhood attachment may therefore offset loneliness both by supplying an alternative source of belonging and by linking adolescents to community resources. Whether school and neighborhood attachment offer comparable protection against the long‐term risks of loneliness is an empirical question, which we address by examining the two contexts side by side.

Using data from the National Longitudinal Study of Adolescent to Adult Health (Add Health), this study examines the association between adolescent loneliness and precarious employment in young adulthood. We conceptualize precarious employment as a multidimensional construct encompassing job stability, material rewards, employment benefits, and workplace autonomy. A key methodological challenge in assessing this relationship is the potential for unobserved family‐level confounding. Family environments shape both adolescents' social experiences and their later socioeconomic trajectories through shared genetic factors, parenting practices, socioeconomic resources, and neighborhood contexts. These unobserved characteristics may simultaneously influence adolescents' propensity to experience loneliness and their likelihood of entering precarious employment in adulthood, leading to biased estimates in conventional regression models that rely solely on observed covariates. To address this concern, we leverage the sibling structure of Add Health and employ sibling fixed effects models that compare siblings within the same family. This approach accounts for all observed and unobserved family‐level characteristics shared by siblings and constant over time, thereby enabling a more rigorous assessment of the association than pooled or cross‐sectional analyses.

Recent research using the same Add Health sibling sample and multidimensional measure of precarious employment found that adolescent depressive symptoms were associated with greater employment precarity, partly through persistent depressive symptoms and lower educational attainment (Kim et al., 2026). However, this research has not examined whether the subjective experience of relational inadequacy is associated with employment precarity beyond broader depressive symptomatology, or whether attachment to adolescent institutional contexts conditions this association. The present study addresses these questions by examining the within‐family association between adolescent loneliness and employment precarity and by comparing school and neighborhood attachment as potential moderators. By integrating a life‐course framework that distinguishes subjective loneliness from the structural properties of social networks, this study advances a more comprehensive understanding of how adolescent social experiences are associated with employment quality in early adulthood. Moreover, by examining the moderating roles of school and neighborhood attachment, the findings highlight the importance of institutional and contextual resources in shaping the long‐term implications of adolescent loneliness. These contributions extend existing research on employment precarity by situating its origins earlier in the life‐course and clarifying the perceptual dimension of social experience through which those origins operate.

## DATA AND METHOD

### Data

This study draws on data from the National Longitudinal Study of Adolescent to Adult Health (Add Health), a nationally representative longitudinal survey designed to examine the health and developmental trajectories of adolescents in the United States. Add Health employed a multistage, stratified, school‐based cluster sampling design, selecting 80 schools with probability proportional to enrollment size. The original cohort was enrolled during the 1994–1995 school year and included students in grades 7 through 12 (Wave I), with follow‐up surveys conducted in 1996 (Wave II), 2001–2002 (Wave III), and 2008–2009 (Wave IV). Together, these waves capture critical life‐course stages, ranging from adolescence to early adulthood and young adulthood. The study collected rich information on participants' social, economic, psychological, cognitive, and physical health, as well as detailed contextual data on family, school, neighborhood, peer, and romantic relationships. Written informed consent was obtained from participants or their parents or caregivers, as appropriate. The present analysis was exempt from full Institutional Review Board review because it relied on de‐identified secondary data.

A key feature of the Add Health study is its inclusion of a large number of sibling pairs. The present analysis was restricted to respondents identified as siblings. The sibling‐subsample consisted of 3703 participants at Wave I, of whom 3105 were successfully followed through Wave IV, when the outcome was assessed. Four respondents with missing outcome data were excluded, yielding a final analytic sample of 3101 individuals. Table S1 in the Supporting Information presents standardized differences in covariate means between the analytic sample and the excluded sample (*N* = 602). Several characteristics differed significantly between the two groups: excluded respondents were slightly older, more likely to be male, Asian, and foreign‐born, less likely to live with two biological parents, and had lower standardized picture vocabulary test (PVT) scores. In contrast, maternal education and family income showed minimal and nonsignificant differences. Importantly, adolescent loneliness—the primary independent variable—did not differ significantly between included and excluded respondents. Although some demographic differences reached statistical significance, most standardized differences were small in magnitude, suggesting limited attrition‐related imbalance and little evidence that selection into the analytic sample was systematically associated with adolescent loneliness.

To address missing data on analysis variables, we employed multiple imputation with chained equations (*m* = 10 imputed datasets) using Stata 17.0 (Allison, 2001). The imputation model conditioned on all analysis variables, wave‐participation indicators, and auxiliary demographic and family covariates, with predictive mean matching for continuous variables, multinomial logistic regression for categorical variables, and logistic regression for binary variables. Although the dependent variable was included in the imputation model, imputed outcome values were not used in the analytic models (von Hippel, 2007). The variables with the highest proportions of missing data were family income (3.3%) and maternal educational attainment (1.4%). Our approach assumes missing at random (MAR) in the sense of Little and Rubin (2019). Although MAR is not directly testable (Enders, 2010), missing completely at random (MCAR) is empirically implausible here because missingness is associated with observed demographic and socioeconomic characteristics (Table S2 in the Supporting Information). Sample retention across Add Health waves is reported in Table S3, with 83.9% of the Wave I sibling frame responding at Wave IV.

### Measures

#### Dependent variable

The outcome variable, precarious employment in adulthood, was assessed at Wave IV (Oddo et al., 2024). Following prior research, we operationalized precarious employment as a multidimensional construct reflecting adverse employment conditions across multiple aspects of the employment relationship (Julià et al., 2017). Eight survey items were used to capture five of the seven commonly identified dimensions: material rewards (total income; access to paid vacation or sick leave), working time arrangements (weekly hours worked), employment stability (job tenure; number of jobs), workers' rights and social protections (employer‐provided retirement benefits; health insurance coverage), and interpersonal relations (decision‐making autonomy at work). Add Health did not contain information necessary to measure the remaining dimensions of collective organization, training, and employability. Each dimension was represented by one or two dichotomous indicators, coded such that higher values reflected more precarious conditions. The indicators were summed to create an index ranging from 0 (least precarious) to 5 (most precarious). Additional details on the measurement of each dimension are provided in Table S4 in the Supporting Information.

The weighting scheme followed the dimension‐balanced logic of the Employment Precariousness Scale (EPRES) tradition (Benach et al., 2014; Vives et al., 2010). Specifically, dimensions measured by a single item (working time arrangements; interpersonal relations) contribute 1 point, while each of the two items within dimensions measured by two items (material rewards; employment stability; workers' rights) contributes 0.5 point, so that every one of the five available dimensions contributes at most 1 point to the overall index (range 0–5). This arrangement prevents dimensions represented by more items from mechanically dominating the composite. In a sensitivity analysis reported in the Supporting Information (Table S5), we re‐estimated all main and moderation models using an equal‐weighted index that sums all eight binary items (range 0–8); substantive conclusions are unchanged.

#### Independent variable

Loneliness, reflecting adolescents' subjective perceptions of social disconnection, was measured at Wave I using a combined set of items drawn from two established traditions of loneliness measurement (Goosby et al., 2013). The first set, consisting of frequency‐of‐feeling items adapted from the Center for Epidemiologic Studies Depression (CES‐D) scale (Radloff, 1977)—an instrument validated extensively in both adolescent and adult populations—asked respondents how often, during the past 7 days, they experienced feeling lonely, feeling disliked by others, and perceiving others as unfriendly, using a 4‐point scale ranging from 0 (“never or rarely”) to 3 (“most or all of the time”). The second set, consisting of perceived‐acceptance items, reflects the affective‐relational tradition of loneliness measurement developed by Perlman and Peplau (1981) and elaborated in contemporary work by Hawkley and Cacioppo (2010) and Cacioppo and Patrick (2008); participants indicated their level of agreement with statements reflecting feelings of being loved and wanted and of social acceptance, rated on a 5‐point scale from 1 (“strongly agree”) to 5 (“strongly disagree”). The five items were combined using factor analysis to derive factor scores, with higher values indicating greater loneliness.

Within the analytic sibling sample, the resulting scale demonstrated acceptable internal consistency (Cronbach's *α* = 0.69). A principal component analysis further revealed two correlated factors (eigenvalues 2.25 and 1.04, cumulatively explaining 65.8% of variance) that correspond to the scale's two source traditions: one factor is defined by the two perceived‐acceptance items (varimax‐rotated loadings 0.82 for “socially accepted” and 0.87 for “loved and wanted”), and the other factor is defined by two of the CES‐D items (varimax‐rotated loadings 0.86 for “people were unfriendly to you” and 0.80 for “felt people disliked you”); the remaining CES‐D item (“felt lonely”) loads approximately equally on both factors (0.45 and 0.44), consistent with a bridging function of the affective “lonely” experience between the two subdimensions of the composite. This pattern supports the discriminant validity of the scale as a measure of subjective social disconnection that is distinct from, though empirically related to, the broader construct of depressive symptoms. We interpret the combined factor score as a theoretically coherent multidimensional summary of adolescents' subjective social disconnection.

#### Moderating variable

School attachment was assessed using three items asking whether, during the past year, respondents felt close to people at school, felt a sense of belonging at school, and felt happy to be at school. Responses were recorded on a 5‐point Likert scale ranging from 1 (“strongly disagree”) to 5 (“strongly agree”) and summed to create a composite score ranging from 3 to 15, with higher values indicating stronger school attachment. The three items are drawn directly from the Add Health In‐Home Questionnaire and constitute a widely used measure of adolescent school connectedness in the developmental literature (Libbey, 2004; McNeely et al., 2002; McNeely & Falci, 2004; Resnick et al., 1997). Within the analytic sibling sample, Cronbach's *α* = 0.86, and a principal component analysis indicates a dominant single factor (eigenvalue = 2.35, 78.2% of variance; loadings 0.87, 0.91, 0.87), supporting the unidimensionality of the composite.

Neighborhood attachment was measured using respondents' perceptions of neighborhood connectedness. Participants indicated whether the following statements were true for them: knowing most people in the neighborhood, having stopped to talk with a neighbor in the past month, and perceiving that people in the neighborhood look out for one another. These dichotomous items were summed to create a scale ranging from 0 to 3, with higher scores reflecting greater neighborhood attachment (Wright & Fitzpatrick, 2006; Zimmerman et al., 2016). The three neighborhood items align conceptually with the neighborhood cohesion and collective efficacy tradition established by Sampson et al. (1997) and have been used in Add Health‐based analyses of adolescent neighborhood context (Browning et al., 2004; Haynie & South, 2005). Within the analytic sibling sample, Cronbach's *α* = 0.73, and a principal component analysis confirms a unidimensional structure (eigenvalue = 1.97, 65.7% of variance; loadings 0.87, 0.85, 0.70). Both school and neighborhood attachment measures were standardized to facilitate interpretation.

#### Control variable

Because demographic characteristics are well‐established correlates of both loneliness and labor market outcomes, the analyses adjusted for a set of individual‐level covariates measured at Wave I. These included age, gender, race/ethnicity (White, Black, Hispanic, Asian, and Other), foreign‐born status, birth order (first‐born), and cognitive ability, measured using the Peabody PVT. We included PVT as a prognostic covariate because cognitive skills are well‐established predictors of educational and occupational attainment and may therefore account for variation in access to stable, protected, and higher‐quality employment (Abrassart, 2013; Heckman, 2006). PVT scores were standardized to have a mean of zero and a standard deviation of one to facilitate interpretation. The models also controlled for family background characteristics, including maternal educational attainment (years of schooling), family income (scaled in $100,000 units), co‐residence with two biological parents, number of siblings, and rural versus nonrural residence. However, family‐level characteristics were excluded from the sibling fixed effects models, which serve as the primary analytic framework for this study to account for unobserved shared familial confounding.

### Analytic strategy

To assess the association between adolescent loneliness and precarious employment in young adulthood, we first estimated ordinary least squares (OLS) regression models. The baseline specification is given by:

(1) $$y_{i}=\beta_{0}+\beta_{1}{\mathrm{AL}}_{i}+X_{i}\delta +Z_{i}\gamma +\varepsilon_{i}$$

where $y_{i}$ denotes precarious employment measured at Wave IV, and ${\mathrm{AL}}_{i}$ represents adolescent loneliness measured at Wave I. The error term $\varepsilon_{i}$ captures unobserved influences on precarious employment not accounted for in the model. To mitigate potential confounding, the model includes a vector of individual‐level covariates ($X_{i}$) and a vector of family‐level covariates ($Z_{i}$), which adjust for observable characteristics related to both adolescent loneliness and later employment outcomes.

A key limitation of the OLS approach is its reliance on the assumption that all relevant confounders are observed. If unmeasured family‐level characteristics are correlated with both adolescent loneliness and precarious employment, OLS estimates may be biased. To address this concern, we estimated sibling fixed effects models that account for unobserved family‐level heterogeneity:

(2) $$y_{\text{is}}=\beta_{0}+\beta_{1}{\mathrm{AL}}_{\text{is}}+X_{\text{is}}\delta +\theta_{s}+\varepsilon_{\text{is}}$$

where $\theta_{s}$ represents sibling fixed effects, capturing all characteristics shared by siblings within the same family, including genetic factors, parental characteristics, socioeconomic background, and shared early‐life environments. Family‐level covariates are omitted from this specification because they are absorbed by the fixed effects, and robust standard errors are clustered at the family‐level to account for within‐family dependence. By exploiting within‐family variation in loneliness between siblings raised in the same household, the sibling fixed‐effects design absorbs all characteristics that are constant across siblings and over time, including genetic endowment, parental socialization practices, and shared early‐life environments. This approach substantially reduces the threat of between‐family confounding. We nonetheless interpret estimates as within‐family associations rather than causal effects, because sibling‐specific unobserved factors may still differ between siblings in ways correlated with both loneliness and later employment outcomes.

To examine whether school and neighborhood attachment (${\mathrm{Att}}_{\text{is}}$) moderates the association between adolescent loneliness and precarious employment, we introduced an interaction (${\mathrm{AL}}_{\text{is}}\times {\mathrm{Att}}_{\text{is}}$) into the model:

(3) $$y_{\text{is}}=\beta_{0}+\beta_{1}{\mathrm{AL}}_{\text{is}}+\beta_{2}{\mathrm{Att}}_{\text{is}}+\beta_{3}{\mathrm{AL}}_{\text{is}}\times {\mathrm{Att}}_{\text{is}}+X_{\text{is}}\delta +\theta_{s}+\varepsilon_{\text{is}}$$

In this equation, $\beta_{3}$ captures the extent to which school and neighborhood attachment moderates the relationship between adolescent loneliness and precarious employment.

Although we present OLS models (Equation 1) alongside sibling fixed‐effects models (Equations 2 and 3) for methodological transparency, the sibling fixed‐effects specification is our preferred inferential framework. The full‐sample and sibling sample OLS models adjust for observed individual‐ and family‐level characteristics, including first‐born status and number of siblings, and allow us to assess whether the estimated association changes when the analysis is restricted to the sibling sample. The sibling fixed‐effects model then compares siblings within the same family and absorbs all observed and unobserved family‐level characteristics shared by siblings, such as sibship size. We therefore anchor substantive interpretation in the sibling FE estimates, treating the OLS comparisons as a descriptive benchmark that makes visible how the association changes as observed and then unobserved family‐level confounding are progressively addressed.

## RESULTS

Table 1 presents descriptive statistics for the analytic sibling sample. On average, respondents reported a precarious employment score of 1.41 in young adulthood (SD = 0.92), with values ranging from 0 to 5. Adolescent loneliness, measured at Wave I, had a mean close to zero (*M* = 0.02, SD = 0.82). The moderating variables—school attachment and neighborhood attachment—were standardized, with means near zero and standard deviations close to one. The average age at baseline was 16.1 years (SD = 1.69), and the sample was nearly evenly split by gender (49% male). The racial/ethnic composition was predominantly White (58%), followed by Black (20%), Hispanic (14%), Asian (6%), and other racial/ethnic groups (1%). Approximately 7% of respondents were foreign‐born, and 31% were first‐born children. Cognitive ability, measured by the standardized PVT score, was centered around zero. Regarding family background, mothers had an average of 13.1 years of education, 60% of respondents lived with two biological parents, and the average number of siblings was 2.24. About 27% of respondents resided in rural areas.

**TABLE 1:** Descriptive statistics of the analytic sibling sample (*N* = 3101).

	Mean/prop.	SD	Min	Max
Dependent variable				
Precarious employment in young adulthood	1.41	0.92	0.00	5.00
Independent variable				
Adolescent loneliness	0.02	0.82	−1.04	4.61
Moderating variable				
School attachment	−0.01	0.99	−0.81	2.21
Neighborhood attachment	0.04	0.99	−3.14	1.45
Individual‐level covariates				
Age	16.10	1.69	12.00	21.00
Male	0.49	0.50	0.00	1.00
White	0.58	0.49	0.00	1.00
Black	0.20	0.40	0.00	1.00
Hispanic	0.14	0.35	0.00	1.00
Asian	0.06	0.24	0.00	1.00
Others	0.01	0.11	0.00	1.00
Foreign‐born status	0.07	0.26	0.00	1.00
First‐born status	0.31	0.46	0.00	1.00
Standardized PVT score	−0.00	0.94	−5.58	3.06
Family‐level covariates				
Maternal education	13.09	2.40	0.00	17.00
Family income	0.46	0.51	0.00	9.99
Co‐residence of two biological parents	0.60	0.49	0.00	1.00
Number of siblings	2.24	1.50	0.00	13.00
Rural status	0.27	0.44	0.00	1.00
*N*	3101			

*Note*: Summary statistics contain imputed values.

Abbreviation: PVT, picture vocabulary test.

Before turning to the regression results, we report pairwise correlations and multicollinearity diagnostics among the three focal constructs. Within the analytic sibling sample, the correlations are: loneliness with school attachment, *r* = −0.37; loneliness with neighborhood attachment, *r* = +0.17; and school attachment with neighborhood attachment, *r* = −0.18. These correlations are reported in Table S6 in the Supporting Information. The magnitudes indicate that, while the three constructs are empirically related, each retains substantial independent variance: for example, loneliness and school attachment share only about 14% of their variance. Variance inflation factors in both the OLS specification and a within‐family demeaned specification corresponding to the sibling fixed‐effects estimator are uniformly modest (all VIFs ≤1.30; mean VIFs 1.12–1.20), well below conventional thresholds of concern (5 or 10). We therefore proceed with confidence that multicollinearity does not meaningfully compromise the precision or interpretation of the regression estimates.

Table 2 presents estimates of the association between adolescent loneliness and precarious employment in young adulthood across three model specifications. Model (1) reports results from the full‐sample using OLS regression with both individual‐ and family‐level covariates, showing that higher levels of adolescent loneliness are significantly associated with greater precarious employment in adulthood (*β* = 0.079, *p* < .001). Model (2) restricts the analysis to the sibling sample and includes the same set of covariates; the estimated association remains positive and statistically significant and is slightly larger in magnitude (*β* = 0.097, *p* < .001). Model (3) further strengthens the design by incorporating sibling fixed effects, thereby accounting for all shared family‐level characteristics. Even under this more stringent specification, adolescent loneliness continues to be associated with higher levels of precarious employment (*β* = 0.110, *p* < .001).

**TABLE 2:** Associations between adolescent loneliness and precarious employment in young adulthood.

	(1)	(2)	(3)
	Precarious employment	Precarious employment	Precarious employment
Sample	Total	Sibling	Sibling
Individual‐level covariates	Yes	Yes	Yes
Family‐level covariates	Yes	Yes	No
Sibling fixed effects	No	No	Yes
Adolescent loneliness	0.079***	0.097***	0.110***
	(0.009)	(0.020)	(0.031)
*N*	15,652	3101	3101

*Note*: Models 1 and 2 present OLS estimates from the full Wave I In‐Home sample and the sibling analytic subsample, respectively; Model 3 presents sibling fixed‐effects estimates, the preferred specification, which absorbs all family‐level characteristics shared by siblings. Individual‐level covariates include age, gender, race/ethnicity, foreign‐born status, first‐born child, and standardized PVT score. Family‐level covariates include mother's education, family income, co‐residence of both biological parents, number of siblings, and rural status. In sibling fixed effects models, robust standard errors are clustered at the family‐level.

*** *p* < .001.

The adolescent loneliness coefficient is stable across the three specifications, increasing modestly from the full‐sample OLS (Model 1) to the sibling‐subsample OLS (Model 2) and again from the sibling‐subsample OLS (Model 2) to the sibling fixed‐effects specification (Model 3). This monotonic increase does not by itself rule out family‐level confounding as a source of the estimated association; among other possibilities, the pattern is consistent with an amplification of measurement‐error attenuation bias when between‐family variation is removed and estimation relies on the more error‐prone within‐family variation. Within the identifying assumptions of the sibling fixed‐effects design, however, the association between adolescent loneliness and precarious employment persists after all family‐level characteristics shared by siblings have been absorbed, so the association is not fully accounted for by shared family confounding.

Table 3 disaggregates precarious employment into specific job‐related dimensions to examine how adolescent loneliness is associated with distinct components of employment precarity. The results indicate substantial heterogeneity across precarity items. Adolescent loneliness is significantly associated with a higher likelihood of lacking paid time off (*β* = 0.032, *p* < .05) and with fewer weeks employed (*β* = 0.041, *p* < .01), even in models that account for sibling fixed effects. Loneliness is also positively associated with the absence of employer‐provided retirement plans (*β* = 0.046, *p* < .01) and with reduced autonomy in workplace decision‐making (*β* = 0.035, *p* < .001). In contrast, no statistically significant associations are observed for relative wages, total work hours, number of jobs, or health insurance coverage once shared family background is controlled. Taken together, these findings suggest that adolescent loneliness is more consistently related to dimensions of employment precarity involving job stability, employment benefits, and workplace control, rather than earnings or workload intensity.

**TABLE 3:** Associations between adolescent loneliness and specific dimensions of precarious employment in young adulthood.

Sample	(1)	(2)	(3)	(4)	(5)	(6)	(7)	(8)
	Sibling	Sibling	Sibling	Sibling	Sibling	Sibling	Sibling	Sibling
Individual‐level covariates	Yes	Yes	Yes	Yes	Yes	Yes	Yes	Yes
Family‐level covariates	Yes	No	No	No	No	No	No	No
Sibling fixed effects	Yes	Yes	Yes	Yes	Yes	Yes	Yes	Yes
Precarity item	Relative wages	Paid time off	Total hours	Weeks employed	Number of jobs	Health insurance	Retirement plan	Freedom to make decisions
Adolescent loneliness	0.027	0.032*	0.002	0.041**	0.000	0.025	0.046**	0.035***
	(0.014)	(0.015)	(0.016)	(0.016)	(0.012)	(0.015)	(0.016)	(0.010)
*N*	3101	3101	3101	3101	3101	3101	3101	3101

*Note*: Individual‐level covariates include age, gender, race/ethnicity, foreign‐born status, first‐born child, and standardized PVT score. Family‐level covariates include mother's education, family income, co‐residence of both biological parents, number of siblings, and rural status. In sibling fixed effects models, robust standard errors are clustered at the family‐level.

* *p* < .05;

** *p* < .01;

*** *p* < .001.

Table 4 examines whether school and neighborhood attachment moderate the association between adolescent loneliness and precarious employment in young adulthood. In Model (1), adolescent loneliness is positively associated with precarious employment (*β* = 0.066, *p* < .05), whereas higher school attachment is negatively associated with precarious employment (*β* = −0.063, *p* < .05), indicating a protective association. Importantly, the interaction between adolescent loneliness and school attachment is negative and statistically significant (*β* = −0.055, *p* < .05), suggesting that higher levels of school attachment attenuate the association between loneliness and later precarious employment. In contrast, Model (2) shows that neighborhood attachment is not significantly associated with precarious employment, nor does it significantly moderate the association between adolescent loneliness and precarious employment.

**TABLE 4:** Moderating roles of school and neighborhood attachment in the association between adolescent loneliness and precarious employment in young adulthood.

	(1)	(2)
	Precarious employment	Precarious employment
Sample	Sibling	Sibling
Individual‐level covariates	Yes	Yes
Family‐level covariates	No	No
Sibling fixed effects	Yes	Yes
Adolescent loneliness	0.066*	0.114***
	(0.034)	(0.031)
School attachment	−0.063*	
	(0.029)	
Adolescent loneliness × School attachment	−0.055*	
	(0.024)	
Neighborhood attachment		−0.037
		(0.032)
Adolescent loneliness × Neighborhood attachment		−0.010
		(0.030)
*N*	3101	3101

*Note*: Individual‐level covariates include age, gender, race/ethnicity, foreign‐born status, first‐born child, and standardized PVT score. Family‐level covariates include mother's education, family income, co‐residence of both biological parents, number of siblings, and rural status. In sibling fixed effects models, robust standard errors are clustered at the family‐level.

* *p* < .05;

*** *p* < .001.

**FIGURE 1:**
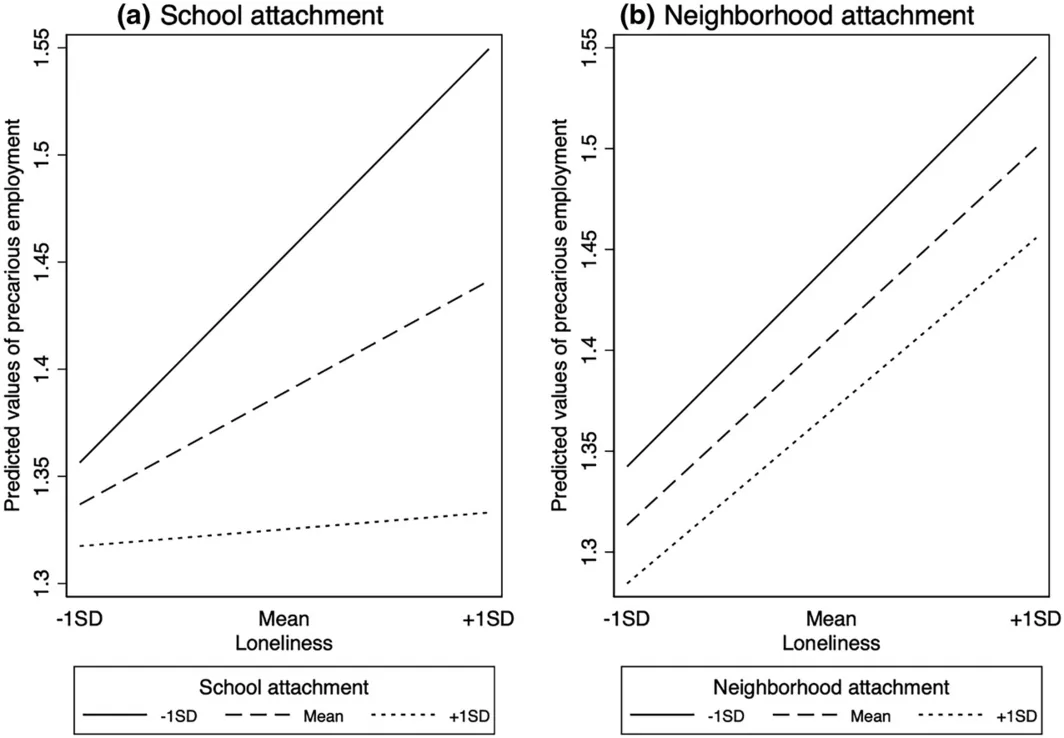
Predicted precarious employment by adolescent loneliness and levels of school and neighborhood attachment.

Figure 1 illustrates predicted values of precarious employment across levels of adolescent loneliness, stratified by school attachment (Panel a) and neighborhood attachment (Panel b). In Panel a, higher levels of adolescent loneliness are associated with greater predicted precarious employment, but the slope of this association varies by school attachment. Specifically, the association between loneliness and precarious employment is steepest among individuals with low school attachment (−1 SD) and substantially attenuated among those with high school attachment (+1 SD), indicating that the positive association between loneliness and precarious employment is weaker among individuals with higher school attachment. In contrast, Panel b shows largely parallel increases in predicted precarious employment across levels of neighborhood attachment, suggesting that neighborhood attachment does not meaningfully moderate the relationship between adolescent loneliness and precarious employment.

To evaluate directly whether adolescent loneliness carries information about precarious employment beyond broader depressive symptomatology, we re‐estimated the three specifications in Table 2 with an additional covariate: a standardized composite of eight nonoverlapping Wave I CES‐D items following the operationalization of adolescent depressive symptoms used by Kim et al. (2026), from which the single item shared with our loneliness scale (“you felt that people disliked you”) was excluded. Adjustment for adolescent depressive symptoms attenuates the loneliness coefficient by only approximately 10% in the sibling fixed‐effects specification (from *β* = 0.110, *p* < .001, to *β* = 0.099, *p* < .01), and loneliness remains highly significant. The same pattern is reproduced in the two OLS specifications: adolescent loneliness remains significant. Moreover, re‐estimating both moderation specifications with the nonoverlapping depression composite as an additional covariate leaves the loneliness × school attachment interaction statistically significant (*β* = −0.053, *p* < .05), and the null pattern for neighborhood attachment moderation is likewise reproduced. These auxiliary specifications, reported in Table S7 of the Supporting Information, indicate that our estimates reflect the labor‐market imprint of adolescent loneliness as a subjective‐relational experience rather than an unmodeled depression pathway.

## DISCUSSION

This study examined the association between adolescent loneliness and precarious employment in young adulthood, with particular attention to the moderating roles of school and neighborhood attachment. Using longitudinal data from Add Health and leveraging sibling fixed effects models to account for shared family‐level confounding, we found that adolescent loneliness is associated with an increased risk of precarious employment in young adulthood. This association persisted even after controlling for observed individual characteristics and unobserved family background, suggesting that subjective social disconnection during adolescence may have enduring implications for employment quality later in life. Moreover, we found evidence that school attachment, but not neighborhood attachment, attenuates the association between adolescent loneliness and subsequent employment precarity, highlighting the importance of institutional contexts in shaping the long‐term consequences of adolescent social experiences.

Before interpreting the substantive findings, we consider an alternative explanation for the observed buffering role of school attachment: that it reflects shared variance or construct overlap with loneliness rather than a genuine buffering process. We acknowledge that loneliness and school attachment, as measured at Wave I, are partially overlapping indicators of adolescents' social–emotional experience within the school context (*r* = −0.37). Several lines of evidence nonetheless argue against a purely construct overlap interpretation. First, the interaction term remains statistically significant in the sibling fixed‐effects specification, which holds constant all family‐level influences shared by siblings; a mechanical artifact of measurement overlap would be less likely to survive this adjustment. Second, neighborhood attachment, which is only weakly correlated with loneliness (*r* = +0.17), does not show a parallel moderating pattern; if shared measurement variance were the mechanical driver of significant interactions, we would expect the weakly correlated moderator to also exhibit some moderation. Third, the substantive pattern—the loneliness–precarious employment association being attenuated among adolescents with higher school attachment—aligns with a social‐institutional buffering interpretation rather than a statistical artifact. Finally, we note that school attachment and neighborhood attachment are themselves only modestly and in fact negatively correlated (*r* = −0.18), indicating that the two contexts do not operate as a single, undifferentiated social sphere in this sample; the observed moderating pattern is therefore a property of one specific institutional context (schools), not of adolescent social embeddedness in general.

The finding that adolescent loneliness is associated with higher levels of precarious employment extends prior research on the long‐term consequences of loneliness. Previous studies have documented associations between loneliness in adolescence and a range of adverse outcomes in adulthood, including poorer mental health, lower educational attainment, and compromised physical health, even after accounting for family background and co‐occurring mental health conditions (Cacioppo et al., 2015; Goosby et al., 2013; Qualter et al., 2015). Our findings add to this literature by demonstrating that adolescent loneliness is also linked to the quality of labor market outcomes, rather than employment status or earnings alone. By focusing on a multidimensional measure of employment precarity, this study highlights that the consequences of adolescent loneliness may be particularly pronounced in domains related to job stability, access to employment‐based benefits, and workplace autonomy—dimensions that have been shown to carry important implications for economic security and health (Benach et al., 2014; Kalleberg, 2009).

The observed association is consistent with life‐course perspectives, which emphasize adolescence as a critical period for the accumulation of psychosocial and institutional resources that shape later socioeconomic outcomes (Elder et al., 2003; Heckman, 2006). Importantly, our use of a subjective measure of loneliness directs attention to perceptual and psychological pathways rather than to the structural resources of an adolescent's social network. Persistent feelings of relational inadequacy may lower relational self‐efficacy and heighten sensitivity to social threat, orientations that can shape how young people approach interpersonally demanding work (Hawkley & Cacioppo, 2010). These subjective experiences may also accompany weaker educational engagement and attainment (Crosnoe, 2011), leaving lonely adolescents with thinner credentials as they enter an increasingly stratified labor market (Kerckhoff, 2001). Although this study did not directly test these mediating mechanisms, the pattern of results, particularly the stronger associations with dimensions of employment stability and workplace control, suggests that adolescent loneliness may shape the kinds of positions individuals are able to obtain rather than simply whether they are employed (Benach et al., 2014).

Another key finding of this study is the finding that school attachment, but not neighborhood attachment, moderates the association between adolescent loneliness and precarious employment. This pattern is consistent with the view that school attachment offsets the very deficits through which loneliness operates. A strong sense of belonging at school can supply supportive ties to teachers and other adults and a basis for competence and self‐efficacy that does not hinge on the peer acceptance lonely adolescents feel they lack, thereby sustaining the educational engagement and relational confidence that loneliness might otherwise erode (Bond et al., 2007; Kwon, Jang, et al., 2025; McNeely & Falci, 2004). Neighborhood attachment, by contrast, did not buffer the association. Although cohesive neighborhoods provide informal support and a sense of communal belonging (Sampson et al., 1997), these resources may be more distal to the specific competencies and institutional pathways that channel adolescents toward stable employment. This contrast hints that the protective role we observe may be specific to schools rather than a general feature of adolescent institutional belonging.

Two further observations bear on the interpretation of these findings. First, because the loneliness scale employed here is a subjective evaluation of perceived social disconnection rather than a measure of structural network position, our estimates speak to the long‐term association between perceived relational adequacy and employment precarity rather than to the role of network size, density, or resource access. A productive direction for future research would be to jointly model subjective loneliness and objective network features to disentangle the relative contributions of the perceptual and structural dimensions of adolescent social life to adult labor‐market outcomes. Although Add Health includes structural social network measures at Wave I—most notably friend nominations from the In‐School Questionnaire and peer‐rejection items—we do not incorporate them in the present analysis. These measures are available only for the saturated‐school subsample, and including them would substantially reduce our sibling analytic sample and undermine statistical power for the sibling fixed‐effects design. Second, because a psychometrically comparable loneliness measure is not available at Wave IV, we cannot decompose the observed association into prior‐adolescent versus concurrent adult components; our estimates should therefore be read as capturing the long‐term imprint of adolescent loneliness on young‐adult employment rather than contemporaneous associations. Future data collections with repeated, comparable loneliness measures across the adolescent‐to‐adult transition would enable finer‐grained decomposition of these pathways.

An additional observation from the correlations reported in Table S6 warrants comment. Adolescent loneliness is modestly and positively correlated with neighborhood attachment (*r* = +0.17), whereas school and neighborhood attachment are modestly and negatively correlated (*r* = −0.18). These patterns are stable across rural and nonrural strata and after adjustment for demographic and socioeconomic characteristics, suggesting that loneliness, school attachment, and neighborhood attachment tap analytically distinct rather than overlapping dimensions of adolescent social life. Loneliness reflects a subjective evaluation of the adequacy of one's own relationships, whereas neighborhood attachment reflects perceived neighborhood connectedness. Drawing on the bonding‐versus‐bridging distinction (Putnam, 2000), dense and homogeneous neighborhood ties may operate as a source of normative pressure that coexists with—rather than alleviates—subjective loneliness. The modest negative correlation between school and neighborhood attachment further suggests that these two contexts function as partially substitutable arenas of adolescent belonging. Consistent with this substantive interpretation, the moderating pattern reported above is specific to school attachment; neighborhood attachment does not moderate the loneliness–precarity association.

The present study extends an emerging line of research on how distinct adolescent risk factors contribute to precarious employment. Kim et al. (2026) show that adolescent depressive symptoms are associated with greater employment precarity, partly through persistent depression and lower educational attainment. Jang et al. (2026) suggest that adolescent juvenile delinquency is linked to more precarious employment outcomes, with education and incarceration jointly accounting for about a quarter of the association. The present study introduces subjective‐relational disconnection—loneliness—as a conceptually distinct pathway, and its incremental contribution is threefold. First, in the supplementary analysis reported above, loneliness carries information about later employment quality that is not reducible to depressive symptomatology. Second, whereas Jang et al. (2026) examine institutional mediation—how schooling and incarceration channel adolescent behavior into adult outcomes—we introduce institutional moderation: whether attachment to schools and neighborhoods attenuates the labor‐market association of a subjective adolescent risk. Third, the finding that school (but not neighborhood) attachment buffers this association, and survives depression adjustment, identifies a specific institutional target additive to the mediation‐based targets of prior work.

Several limitations should be acknowledged. First, although selection into the analytic sibling sample was not systematically related to either moderator (standardized mean differences vs. excluded cases: 0.04 for school attachment, 0.06 for neighborhood attachment; both n.s.) or to adolescent loneliness (standardized difference − 0.07, n.s.), it was associated with demographic characteristics such as age, gender, foreign‐born status, race/ethnicity, and co‐residence with both biological parents. Generalization of the moderation findings to populations differing substantially on these demographic dimensions should therefore be approached with appropriate caution. Second, the loneliness battery used at Wave I was not replicated in a comparable form at Wave IV, precluding analyses that would jointly identify the unique contributions of adolescent versus concurrent adult loneliness. Likewise, school attachment, as conceptualized here, is inherently tied to the adolescent school context and has no psychometrically equivalent Wave IV counterpart. These measurement asymmetries are a limitation of the Add Health instrument rather than of the present analytic choices, and they point to a priority for future longitudinal data collections on loneliness and social belonging.

Third, Add Health did not collect items permitting valid operationalization of two commonly cited dimensions of precarious employment—collective organization (e.g., union coverage) and training and employability—and our index therefore captures five of the seven dimensions identified in the contemporary precarity literature. A sensitivity analysis using an equal‐weighted index yielded substantively identical conclusions, but the omission of these two dimensions may nonetheless attenuate the measured precariousness of some respondents. Fourth, the Add Health cohort reached young adulthood at Wave IV in 2008–2009, before the widespread adoption of social media, the COVID‐19 pandemic, and more recent changes in labor‐market organization. Although Add Health remains the most recent dataset suited to examining this question using a sibling fixed‐effects design, the observed associations may not generalize unchanged to contemporary adolescents or to subsequent health outcomes. Future research using more recent data may wish to examine the replicability of these findings.

The findings of this study underscore the importance of situating employment precarity within a life‐course framework that considers the potential relevance of adolescent social experiences for later employment quality. Although employment precarity in young adulthood is often understood in relation to macroeconomic conditions and labor‐market institutions, our results suggest that it may also be associated with earlier social and developmental experiences (Schoon & Bynner, 2003). In particular, the weaker association between loneliness and employment precarity among adolescents with stronger school attachment identifies school belonging as a potentially important context for future intervention research. Contemporary applications, however, should recognize that adolescents' social relationships now span both online and in‐person settings and that the meaning of school attachment may have changed following the widespread adoption of social media and the COVID‐19 pandemic. School‐based initiatives that foster belonging, supportive student‐teacher relationships, and inclusive climates, therefore, warrant further evaluation as possible approaches to reducing longer‐term socioeconomic vulnerability among socially isolated adolescents (Durlak et al., 2011; Roorda et al., 2011).

## AUTHOR CONTRIBUTIONS

**Hyewon Son:** Methodology; investigation; writing – original draft; writing – review and editing; formal analysis. **Kyungeun Song:** Investigation; methodology; writing – original draft; writing – review and editing; formal analysis. **Jinho Kim:** Supervision; formal analysis; conceptualization; methodology; writing – original draft; investigation; writing – review and editing.

## FUNDING INFORMATION

The authors received no financial support for the research, authorship, and/or publication of this article.

## CONFLICT OF INTEREST STATEMENT

The authors declared no potential conflicts of interest with respect to the research, authorship, and/or publication of this article.

## ETHICS STATEMENT

As this study involved secondary analysis of anonymized data, it was exempt from full review by an institutional review board (KUIRB‐2022‐0032).

## PATIENT CONSENT STATEMENT

All participants (or their next of kin/caregiver) provided written informed consent in accordance with Add Health study protocols.

## Supporting information


**Table S1.** Standardized difference in covariate means for the included (*N* = 3101) and excluded samples (*N* = 602).
**Table S2.** Comparison of analytic sample cases with and without imputed values (observed values only).
**Table S3.** Sample retention across Add Health waves in the sibling analytic frame.
**Table S4.** Precarious employment dimensions and indicators used in the precarious employment index.
**Table S5.** Sensitivity analysis: sibling fixed‐effects estimates using an equal‐weighted precarious employment index.
**Table S6.** Correlations and variance inflation factors among key constructs.
**Table S7.** Sibling fixed‐effects estimates of adolescent loneliness on precarious employment, with and without a nonoverlapping depression covariate.

## Data Availability

The data that support the findings of this study are available from the Carolina Population Center. Restrictions apply to the availability of these data, which were used under license for this study. Data are available at https://data.cpc.unc.edu/ with the permission of the Carolina Population Center.

## References

[jora70266-bib-0001] Abrassart, A. (2013). Cognitive skills matter: The employment disadvantage of low‐educated Workers in Comparative Perspective. European Sociological Review, 29(4), 707–719. 10.1093/esr/jcs049

[jora70266-bib-0002] Allison, P. D. (2001). Missing Data. Sage Publications.

[jora70266-bib-0003] Benach, J. , Vives, A. , Amable, M. , Vanroelen, C. , Tarafa, G. , & Muntaner, C. (2014). Precarious employment: Understanding an emerging social determinant of health. Annual Review of Public Health, 35(1), 229–253. 10.1146/annurev-publhealth-032013-182500 24641559

[jora70266-bib-0004] Benach, J. , Vives, A. , Tarafa, G. , Delclos, C. , & Muntaner, C. (2016). What should we know about precarious employment and health in 2025? Framing the agenda for the next decade of research. International Journal of Epidemiology, 45(1), 232–238. 10.1093/ije/dyv342 26744486

[jora70266-bib-0005] Benner, A. D. (2011). Latino adolescents' loneliness, academic performance, and the buffering nature of friendships. Journal of Youth and Adolescence, 40(5), 556–567. 10.1007/s10964-010-9561-2 20571900 PMC3033456

[jora70266-bib-0006] Bond, L. , Butler, H. , Thomas, L. , Carlin, J. , Glover, S. , Bowes, G. , & Patton, G. (2007). Social and school connectedness in early secondary school as predictors of late teenage substance use, mental health, and academic outcomes. Journal of Adolescent Health, 40(4), 357.e9–357.e18. 10.1016/j.jadohealth.2006.10.013 17367730

[jora70266-bib-0007] Browning, C. R. , Leventhal, T. , & Brooks‐Gunn, J. (2004). Neighborhood context and racial differences in early adolescent sexual activity. Demography, 41(4), 697–720. 10.1353/dem.2004.0029 15622950

[jora70266-bib-0008] Cacioppo, J. T. , & Patrick, W. (2008). Loneliness: Human nature and the need for social connection. W. W. Norton & Company.

[jora70266-bib-0009] Cacioppo, S. , Grippo, A. J. , London, S. , Goossens, L. , & Cacioppo, J. T. (2015). Loneliness: Clinical import and interventions. Perspectives on Psychological Science, 10(2), 238–249. 10.1177/1745691615570616 25866548 PMC4391342

[jora70266-bib-0010] Coleman, J. S. (1988). Social capital in the creation of human capital. American Journal of Sociology, 94, S95–S120. 10.1086/228943

[jora70266-bib-0011] Crosnoe, R. (2011). Fitting in, standing out: Navigating the social challenges of high school to get an education. Cambridge University Press. 10.1017/CBO9780511793264

[jora70266-bib-0012] Crosnoe, R. , & Johnson, M. K. (2011). Research on adolescence in the twenty‐first century. Annual Review of Sociology, 37(1), 439–460. 10.1146/annurev-soc-081309-150008 PMC5695926 29167597

[jora70266-bib-0013] Donnelly, R. (2022). Precarious work in midlife: Long‐term implications for the health and mortality of women and men. Journal of Health and Social Behavior, 63(1), 142–158. 10.1177/00221465211055090 34794348

[jora70266-bib-0014] Durlak, J. A. , Weissberg, R. P. , Dymnicki, A. B. , Taylor, R. D. , & Schellinger, K. B. (2011). The impact of enhancing students' social and emotional learning: A meta‐analysis of school‐based universal interventions. Child Development, 82(1), 405–432. 10.1111/j.1467-8624.2010.01564.x 21291449

[jora70266-bib-0015] Elder, G. H. , Johnson, M. K. , & Crosnoe, R. (2003). The emergence and development of life course theory. In J. T. Mortimer & M. J. Shanahan (Eds.), Handbook of the life course (pp. 3–19). Springer. 10.1007/978-0-306-48247-2_1

[jora70266-bib-0016] Enders, C. K. (2010). Applied missing data analysis. Guilford Press.

[jora70266-bib-0017] Gash, V. (2008). Bridge or trap? Temporary workers' transitions to unemployment and to the standard employment contract. European Sociological Review, 24(5), 651–668. 10.1093/esr/jcn027

[jora70266-bib-0018] Goosby, B. J. , Bellatorre, A. , Walsemann, K. M. , & Cheadle, J. E. (2013). Adolescent loneliness and health in early adulthood. Sociological Inquiry, 83(4), 505–536. 10.1111/soin.12018 PMC3810978 24187387

[jora70266-bib-0019] Hawkley, L. C. , & Cacioppo, J. T. (2010). Loneliness matters: A theoretical and empirical review of consequences and mechanisms. Annals of Behavioral Medicine, 40(2), 218–227. 10.1007/s12160-010-9210-8 20652462 PMC3874845

[jora70266-bib-0020] Haynie, D. L. , & South, S. J. (2005). Residential mobility and adolescent violence. Social Forces, 84(1), 361–374. 10.1353/sof.2005.0104

[jora70266-bib-0021] Heckman, J. J. (2006). Skill formation and the economics of investing in disadvantaged children. Science, 312(5782), 1900–1902. 10.1126/science.1128898 16809525

[jora70266-bib-0022] Jang, H. , Park, H. , Kim, T. , & Kim, J. (2026). Juvenile delinquency and precarious employment in adulthood: A longitudinal sibling fixed effects analysis of educational and institutional pathways. American Journal of Criminal Justice. 10.1007/s12103-025-09888-7

[jora70266-bib-0023] Julià, M. , Vanroelen, C. , Bosmans, K. , Van Aerden, K. , & Benach, J. (2017). Precarious employment and quality of employment in relation to health and well‐being in Europe. International Journal of Health Services, 47(3), 389–409. 10.1177/0020731417707491 28449605

[jora70266-bib-0024] Kalleberg, A. L. (2009). Precarious work, insecure workers: Employment relations in transition. American Sociological Review, 74(1), 1–22. 10.1177/000312240907400101

[jora70266-bib-0025] Kerckhoff, A. C. (2001). Education and social stratification processes in comparative perspective. Sociology of Education, 74, 3–18. 10.2307/2673250

[jora70266-bib-0026] Kim, J. , Jang, H. , Kwon, K. Y. , & Park, H. (2026). Understanding the link between adolescent depression and precarious employment in adulthood: Evidence from a sibling fixed effects analysis. Journal of Child Psychology and Psychiatry, 67(5), 663–672. 10.1111/jcpp.70047 40897673 PMC13102044

[jora70266-bib-0027] Kim, J. , Kwon, K. Y. , & Sutin, A. R. (2025). Adolescent loneliness and violence in adulthood: Distinguishing between victimization and perpetration in intimate and nonintimate partner violence. Psychology of Violence, 16, 217–226. 10.1037/vio0000625

[jora70266-bib-0028] Kwon, K. Y. , Jang, H. , Subramanian, S. V. , & Kim, J. (2025). School‐based social relationships and children's psychological health: Examining heterogeneity by relationship source and child gender. European Child & Adolescent Psychiatry, 35, 823–833. 10.1007/s00787-025-02873-9 41108395

[jora70266-bib-0029] Kwon, K. Y. , Nam, N. , & Kim, J. (2025). Adolescent loneliness and later‐life depressive symptoms: The intersecting roles of gender and socioeconomic status. Social Science & Medicine, 384, 118588. 10.1016/j.socscimed.2025.118588 40957377

[jora70266-bib-0030] Libbey, H. P. (2004). Measuring student relationships to school: Attachment, bonding, connectedness, and engagement. Journal of School Health, 74(7), 274–283. 10.1111/j.1746-1561.2004.tb08284.x 15493704

[jora70266-bib-0031] Lin, N. (2001). Social Capital. Cambridge University Press. 10.1017/CBO9780511815447

[jora70266-bib-0032] Little, R. , & Rubin, D. (2019). Statistical analysis with missing data (Third ed.). Wiley. 10.1002/9781119482260

[jora70266-bib-0033] Matthews, T. , Qualter, P. , Bryan, B. T. , Caspi, A. , Danese, A. , Moffitt, T. E. , Odgers, C. L. , Strange, L. , & Arseneault, L. (2023). The developmental course of loneliness in adolescence: Implications for mental health, educational attainment, and psychosocial functioning. Development and Psychopathology, 35(2), 537–546. 10.1017/S0954579421001632 35109947 PMC9346093

[jora70266-bib-0034] McNeely, C. A. , & Falci, C. (2004). School connectedness and the transition into and out of health‐risk behavior among adolescents: A comparison of social belonging and teacher support. Journal of School Health, 74(7), 284–292. 10.1111/j.1746-1561.2004.tb08285.x 15493705

[jora70266-bib-0035] McNeely, C. A. , Nonnemaker, J. M. , & Blum, R. W. (2002). Promoting school connectedness: Evidence from the National Longitudinal Study of adolescent health. Journal of School Health, 72(4), 138–146. 10.1111/j.1746-1561.2002.tb06533.x 12029810

[jora70266-bib-0036] Mills, M. C. , & Blossfeld, H.‐P. (2005). Globalization, uncertainty and the early life course: A theoretical framework. In H.‐P. Blossfeld , E. Klijzing , M. Mills , & K. Kurz (Eds.), Globalization, uncertainty and youth in society (pp. 1–24). Routledge.

[jora70266-bib-0037] Oddo, V. M. , Mabrouk, S. , Andrea, S. B. , Ahonen, E. Q. , Winkler, M. R. , Vignola, E. F. , & Hajat, A. (2024). The association between precarious employment and stress among working aged individuals in the United States. Preventive Medicine, 187, 108123. 10.1016/j.ypmed.2024.108123 39216552 PMC11700481

[jora70266-bib-0038] Park, H. , Park, G.‐R. , & Kim, J. (2024). Transitioning into and out of precarious employment and life satisfaction: Evidence from asymmetric fixed effects models. Social Science & Medicine, 341, 116539. 10.1016/j.socscimed.2023.116539 38160611

[jora70266-bib-0039] Perlman, D. , & Peplau, L. A. (1981). Toward a social psychology of loneliness. In S. Duck & R. Gilmour (Eds.), Personal relationships 3: Personal relationships in disorder (pp. 31–56). Academic Press.

[jora70266-bib-0040] Putnam, R. D. (2000). Bowling alone: The collapse and revival of American community. Simon and Schuster.

[jora70266-bib-0041] Qualter, P. , Vanhalst, J. , Harris, R. , Van Roekel, E. , Lodder, G. , Bangee, M. , Maes, M. , & Verhagen, M. (2015). Loneliness across the life span. Perspectives on Psychological Science, 10(2), 250–264. 10.1177/1745691615568999 25910393

[jora70266-bib-0042] Radloff, L. S. (1977). The CES‐D scale. Applied Psychological Measurement, 1(3), 385–401. 10.1177/014662167700100306

[jora70266-bib-0043] Resnick, M. D. , Bearman, P. S. , Blum, R. W. , Bauman, K. E. , Harris, K. M. , Jones, J. , Tabor, J. , Beuhring, T. , Sieving, R. E. , Shew, M. , Ireland, M. , Bearinger, L. H. , & Udry, J. R. (1997). Protecting adolescents from harm findings from the National Longitudinal Study on adolescent health. JAMA, 278(10), 823–832. 10.1001/jama.1997.03550100049038 9293990

[jora70266-bib-0044] Roorda, D. L. , Koomen, H. M. Y. , Spilt, J. L. , & Oort, F. J. (2011). The influence of affective teacher–student relationships on Students' School engagement and achievement. Review of Educational Research, 81(4), 493–529. 10.3102/0034654311421793

[jora70266-bib-0045] Sampson, R. J. , Raudenbush, S. W. , & Earls, F. (1997). Neighborhoods and violent crime: A multilevel study of collective efficacy. Science, 277(5328), 918–924. 10.1126/science.277.5328.918 9252316

[jora70266-bib-0046] Schoon, I. , & Bynner, J. (2003). Risk and resilience in the life course: Implications for interventions and social policies. Journal of Youth Studies, 6(1), 21–31. 10.1080/1367626032000068145

[jora70266-bib-0047] Spithoven, A. W. M. , Bijttebier, P. , & Goossens, L. (2017). It is all in their mind: A review on information processing bias in lonely individuals. Clinical Psychology Review, 58, 97–114. 10.1016/j.cpr.2017.10.003 29102150

[jora70266-bib-0048] Stanton‐Salazar, R. D. (2010). A social capital framework for the study of institutional agents and their role in the empowerment of low‐status students and youth. Youth & Society, 43(3), 1066–1109. 10.1177/0044118X10382877

[jora70266-bib-0049] Vives, A. , Amable, M. , Ferrer, M. , Moncada, S. , Llorens, C. , Muntaner, C. , Benavides, F. G. , & Benach, J. (2010). The employment precariousness scale (EPRES): Psychometric properties of a new tool for epidemiological studies among waged and salaried workers. Occupational and Environmental Medicine, 67(8), 548–555. 10.1136/oem.2009.048967 20576923

[jora70266-bib-0050] von Hippel, P. T. (2007). Regression with missing Ys: An improved strategy for analyzing multiply imputed data. Sociological Methodology, 37(1), 83–117. 10.1111/j.1467-9531.2007.00180.x

[jora70266-bib-0051] Wright, D. R. , & Fitzpatrick, K. M. (2006). Social capital and adolescent violent behavior: Correlates of fighting and weapon use among secondary school students. Social Forces, 84(3), 1435–1453. 10.1353/sof.2006.0077

[jora70266-bib-0052] Zimmerman, G. M. , Rees, C. , & Farrell, C. (2016). Contextual determinants of adolescent perceived early fatality. Journal of Youth and Adolescence, 45(8), 1546–1559. 10.1007/s10964-016-0523-1 27325518

