# Supplementary material for: Adolescent loneliness and precarious employment in young adulthood: The moderating role of school attachment
Source: J Res Adolesc. 2026 Sep 10;36(3):e70266. doi: 10.1111/jora.70266 (PMC13559853; doi:10.1111/jora.70266)
Supplement: Supplementary file 1 [file JORA-36-0-s001.docx]

# Supporting Information

## Table S1. Standardized difference in covariate means for the included (N=3101) and excluded samples (N=602)

|  | (1) | (2) |
| --- | --- | --- |
| Variable | Standardized difference | p-value |
| Adolescent loneliness | -0.072 | 0.101 |
| School attachment | 0.035 | 0.432 |
| Neighborhood attachment | 0.061 | 0.178 |
| Age | 0.132 | 0.002 |
| Male | 0.218 | 0.000 |
| White | -0.234 | 0.000 |
| Black | 0.026 | 0.536 |
| Hispanic | 0.133 | 0.002 |
| Asian | 0.201 | 0.000 |
| Others | 0.056 | 0.157 |
| Foreign-born status | 0.230 | 0.000 |
| First-born status | 0.059 | 0.152 |
| Standardized PVT score | -0.178 | 0.000 |
| Maternal education | -0.062 | 0.201 |
| Family income | -0.007 | 0.898 |
| Co-residence of two biological parents | -0.104 | 0.017 |
| Number of siblings | 0.053 | 0.248 |
| Rural status | 0.009 | 0.846 |

*Note*. Standardized differences are calculated as the mean of the excluded sample minus the mean of the included sample.

## Table S2. Comparison of analytic-sample cases with and without imputed values (observed values only)

| Variable | Non-imputed  (N = 2,057) | Imputed  (N = 1,044) | Difference | p-value |
| --- | --- | --- | --- | --- |
| Precarious employment | 1.38 | 1.45 | +0.07 | .038 |
| Adolescent loneliness | −0.03 | +0.12 | +0.15 | <.001 |
| School attachment | +0.06 | 0.00 | −0.06 | .118 |
| Neighborhood attachment | −0.04 | +0.05 | +0.09 | .022 |
| Age | 16.03 | 16.23 | +0.20 | .002 |
| Male | 0.49 | 0.47 | −0.03 | .148 |
| Standardized PVT score | +0.10 | −0.24 | −0.34 | <.001 |
| Maternal education | 13.23 | 12.92 | −0.30 | .003 |
| Family income | 0.47 | 0.41 | −0.06 | .032 |
| Share of analytic sample | 66.3% | 33.7% |  |  |

*Note.* Imputed cases are analytic-sample members for whom at least one analysis variable required imputation; non-imputed cases had complete observations on all analysis variables. Means and t-tests are computed on observed values only (not on imputed values). Differences indicate that missingness is predictable from observed characteristics (e.g., loneliness, PVT, maternal education, family income), which is consistent with the Missing At Random (MAR) assumption and inconsistent with Missing Completely At Random (MCAR). These patterns justify the use of multiple imputation conditional on a rich set of predictors rather than listwise deletion.

## Table S3. Sample retention across Add Health waves in the sibling analytic frame

| Stage | N | % of sibling frame |
| --- | --- | --- |
| Sibling subsample (Wave I) | 3,703 | 100.0% |
| Wave II respondents | 3,393 | 91.6% |
| Wave III respondents | 3,037 | 82.0% |
| Wave IV respondents | 3,105 | 83.9% |
| Analytic sample (W4, non-missing outcome) | 3,101 | 83.7% |

*Note.* The sibling subsample consists of Wave I In-Home respondents with at least one co-residing full biological sibling also enrolled in Add Health. The analytic sample comprises sibling-subsample members who responded at Wave IV and had non-missing adult precarious-employment outcome. Retention from Wave I to Wave IV is 83.9%, indicating modest wave-to-wave attrition.

## Table S4. Precarious employment dimensions and indicators used in the precarious employment index

| **Dimensions** | **Item** | **Score** | **Question(s) in Add Health** |
| --- | --- | --- | --- |
| 1. Material rewards | Relative wages | 0.5 pt.: Total income earned in the past year below the U.S. median  0 pt.: Otherwise | In the last calendar year, how much income did you receive from personal earnings before taxes, that is, wages or salaries, including tips, bonuses, and overtime pay, and income from self-employment? |
|  | Paid time off | 0.5 pt.: No paid time off  0 pt.: Otherwise | Does your employer make the following available to you: paid vacation or sick leave? |
| 2. Work time arrangements | Total hours | 1 pt.: Total hours/week worked <20 or >40  0 pt.: Otherwise | How many hours a week do you usually work at this job? |
| 3. Stability | Weeks employed | 0.5 pt.: Number of weeks worked/employed in past calendar year is <48 weeks  0 pt.: ≥48 weeks | In what month and year did you begin your current primary job? |
|  | Number of jobs | 0.5 pt.: Number of jobs held >1  0 pt.: Otherwise | On how many jobs are you currently working for pay at least 10 hours a week? |
| 4. Workers’ rights | Health insurance | 0.5 pt.: Health insurance not offered by employer  0 pt.: Otherwise | Does your employer make the following available to you... health insurance? |
|  | Retirement plan | 0.5 pt.: No retirement plan (other than social security) offered by the employer  0 pt.: Otherwise | Does your employer make the following available to you... benefits (such as 401k, 403b, or a company pension plan)? |
| 5. Interpersonal relations | Freedom to make decisions | 1 pt.: Minimal freedom to make decisions without supervision  0 pt.: Otherwise | Overall, how often do you have the freedom to make important decisions about what you do at work and how you do it? |

## Table S5. Sensitivity analysis: sibling fixed-effects estimates using an equal-weighted precarious employment index

|  | (1) | (2) | (3) |
| --- | --- | --- | --- |
|  | Precarious employment | Precarious employment | Precarious employment |
| Adolescent loneliness | 0.181*** | 0.111 | 0.187*** |
|  | (0.053) | (0.058) | (0.053) |
| School attachment |  | −0.090 |  |
|  |  | (0.050) |  |
| Adolescent loneliness × School attachment |  | −0.095* |  |
|  |  | (0.041) |  |
| Neighborhood attachment |  |  | −0.065 |
|  |  |  | (0.055) |
| Adolescent loneliness × Neighborhood attachment |  |  | 0.002 |
|  |  |  | (0.052) |
| *N* | 3101 |  | 3101 |

*Note.* Sibling fixed-effects estimates with robust standard errors clustered at the family level (in parentheses). The equal-weighted index sums eight binary precarious-employment items (range 0–8) without the dimension-balancing weights used in the main analysis. All models include age, gender, foreign-born status, first-born status, and standardized PVT score. The substantive conclusions are identical to those from the main analysis: adolescent loneliness is positively associated with precarious employment, school attachment significantly buffers this association, and neighborhood attachment does not. * p < .05, ** p < .01, *** p < .001.

## Table S6. Correlations and variance inflation factors among key constructs

*Panel A. Pairwise correlations*

|  | (1) Loneliness | (2) School attachment | (3) Neighborhood attachment | (4) Depression composite |
| --- | --- | --- | --- | --- |
| (1) Loneliness | 1.000 |  |  |  |
| (2) School attachment | −0.368 | 1.000 |  |  |
| (3) Neighborhood attachment | 0.167 | −0.182 | 1.000 |  |
| (4) Depression composite | 0.351 | −0.143 | 0.058 | 1.000 |

*Note.* Pairwise correlations are computed in the first imputed dataset (m = 1).

*Panel B. Variance inflation factors*

| Specification | Loneliness | School attachment | Neighborhood attachment | Mean VIF |
| --- | --- | --- | --- | --- |
| (a) OLS: main effects | 1.03 | — | — | 1.13 |
| (b) OLS: both moderators | 1.21 | 1.19 | 1.09 | 1.15 |
| (c) OLS: × school interaction | 1.28 | 1.19 | — | 1.15 |
| (d) OLS: × neighbor interaction | 1.07 | — | 1.09 | 1.12 |
| (e) Within-family demeaned (FE-relevant) | 1.02–1.21 | 1.14 | 1.03–1.04 | 1.15–1.20 |

*Note.* VIFs are computed in the first imputed dataset (m = 1). OLS VIFs are from regression specifications including all individual- and family-level covariates. Within-family demeaned VIFs diagnose the multicollinearity structure relevant to the sibling fixed-effects estimator and are computed by regressing family-mean-deviated outcome on family-mean-deviated regressors. For the FE-relevant specification, ranges summarize variation across the four model specifications (a)–(d). All VIFs are well below the conventional thresholds for concern (5 or 10).

## Table S7. Sibling fixed-effects estimates of adolescent loneliness on precarious employment, with and without a non-overlapping depression covariate

*Panel A. Main-effect models corresponding to Table 2*

|  | (1) | (2) | (3) | (1D) | (2D) | (3D) |
| --- | --- | --- | --- | --- | --- | --- |
|  | Precarious employment | Precarious employment | Precarious employment | Precarious employment | Precarious employment | Precarious employment |
| Sample | Total | Sibling | Sibling | Total | Sibling | Sibling |
| Individual-level covariates | Yes | Yes | Yes | Yes | Yes | Yes |
| Family-level covariates | Yes | Yes | No | Yes | Yes | No |
| Sibling fixed effects | No | No | Yes | No | No | Yes |
| Depression composite | No | No | No | Yes | Yes | Yes |
| Adolescent loneliness | 0.079*** | 0.097*** | 0.110*** | 0.074*** | 0.089*** | 0.099** |
|  | (0.009) | (0.020) | (0.031) | (0.010) | (0.021) | (0.033) |
| Depression composite |  |  |  | 0.012 | 0.017 | 0.029 |
|  |  |  |  | (0.008) | (0.018) | (0.027) |
| *N* | 15,652 | 3,101 | 3,101 | 15,634 | 3,097 | 3,097 |

*Panel B. Moderation models corresponding to Table 4*

|  | (1) | (2) | (1D) | (2D) |
| --- | --- | --- | --- | --- |
|  | Precarious employment | Precarious employment | Precarious employment | Precarious employment |
| Sample | Sibling | Sibling | Sibling | Sibling |
| Individual-level covariates | Yes | Yes | Yes | Yes |
| Family-level covariates | No | No | No | No |
| Sibling fixed effects | Yes | Yes | Yes | Yes |
| Depression composite | No | No | Yes | Yes |
| Adolescent loneliness | 0.066* | 0.114*** | 0.057 | 0.103** |
|  | (0.034) | (0.031) | (0.035) | (0.033) |
| School attachment | −0.063* |  | −0.063* |  |
|  | (0.029) |  | (0.029) |  |
| Adolescent loneliness × School attachment | −0.055* |  | −0.053* |  |
|  | (0.024) |  | (0.024) |  |
| Neighborhood attachment |  | −0.037 |  | −0.038 |
|  |  | (0.032) |  | (0.032) |
| Adolescent loneliness × Neighborhood attachment |  | −0.010 |  | −0.010 |
|  |  | (0.030) |  | (0.030) |
| Depression composite |  |  | 0.025 | 0.029 |
|  |  |  | (0.027) | (0.027) |
| *N* | 3,101 | 3,101 | 3,097 | 3,097 |

*Note.* Sibling fixed-effects estimates from Panel A columns (3) and (3D) and all Panel B columns; OLS estimates in Panel A columns (1)-(2) and (1D)-(2D). All models include individual-level covariates (age, gender, race/ethnicity, foreign-born status, first-born status, standardized PVT score); OLS models additionally include family-level covariates (maternal education, family income, co-residence of both biological parents, number of siblings, rural status). The non-overlapping depression composite is a standardized mean of eight Wave I CES-D items following Kim et al.’s (2026) operationalization of adolescent depressive symptoms, excluding the single item (“you felt that people disliked you”) shared with the loneliness scale used in this study. The eight items are: (1) “You were bothered by things that usually do not bother you”; (2) “You could not shake off the blues even with help from your family and your friends”; (3) “You felt that you were just as good as other people” (reverse-coded); (4) “You had trouble keeping your mind on what you were doing”; (5) “You felt depressed”; (6) “You felt that you were too tired to do things”; (7) “You enjoyed life” (reverse-coded); and (8) “You felt sad.” None of these eight items appears in the loneliness scale used in this study, whose three CES-D items are “You felt lonely,” “You thought people were unfriendly to you,” and “You felt that people disliked you.” Standard errors in parentheses; robust standard errors clustered at the family level in sibling fixed-effects models. * p < 0.05; ** p < 0.01; *** p < 0.001.
